# Pneumococcal Vaccination as Cardiovascular and Cardiorespiratory Prevention in Adults with Heart Disease

**DOI:** 10.3390/jcm15218220

**Published:** 2026-10-22

**Authors:** Clara Bonanad, Guillermo Barreres, Nora Garcia-Collado, Daniela Maidana, David Vivas, Sergio Raposeiras, Elena Fortuny, Diego Segura-Rodriguez, Gonzalo Alonso-Salinas, Esther Redondo, Alberto Garcia-Lledó, Sergio García-Blas, Vivencio Barrios

**Affiliations:** 1 Department of Medicine, University of Valencia, 46010 Valencia, Spain; 2 Fundación para la Investigación del Hospital Clínico de la Comunidad Valenciana (INCLIVA), 46010 Valencia, Spain; gbarreres@incliva.es (G.B.); nora.garcia.collado@gmail.com (N.G.-C.); 3 Centro de Investigación Biomédica en Red de Enfermedades Cardiovasculares (CIBERCV), Instituto de Salud Carlos III, 28029 Madrid, Spain; 4 Department of Cardiology, Hospital Clínico Universitario de Valencia, 46010 Valencia, Spain; 5 Department of Cardiovascular Surgery, Hospital Universitario Virgen Macarena, 41042 Sevilla, Spain; danielamaidana@campus.fmed.uba.ar; 6 Department of Cardiology, Hospital Clínico San Carlos, 28040 Madrid, Spain; dvivas@secardiologia.es; 7 Department of Cardiology, Hospital Universitario Álvaro Cunqueiro, 38312 Vigo, Spain; raposeiras26@hotmail.com; 8 Department of Cardiology, Hospital Universitario Son Espases, 07120 Palma de Mallorca, Spain; elena.fortuny@ssib.es; 9 Department of Cardiology, Hospital Universitario Clínico San Cecilio, 18016 Granada, Spain; 10 Instituto de Investigación Biosanitaria ibs.GRANADA, 18012 Granada, Spain; 11 Department of Medicine, University of Granada, 18071 Granada, Spain; 12 Department of Cardiology, Hospital Universitario de Navarra, 31008 Pamplona, Spain; gonzalol.alonso@gmail.com; 13 Health Sciences Department, Universidad Pública de Navarra (UPNA), 31006 Pamplona, Spain; 14 Technical Unit for Vaccination and International Health, Madrid Salud Autonomous Organization, 28007 Madrid, Spain; redondome@madrid.es; 15 Departamento de Cardiología, Hospital Universitario Príncipe de Asturias, 28805 Alcalá de Henares, Spain; alberto.garcia-lledo@uah.es; 16 Department of Cardiology, Hospital Universitario Ramón y Cajal, Universidad de Alcalá, 28034 Madrid, Spain; vivenciobarrios@gmail.com

**Keywords:** *Streptococcus pneumoniae*, pneumococcal vaccination, cardiovascular prevention, heart failure, myocardial infarction, older adults

## Abstract

**Background/Objectives:** *Streptococcus pneumoniae* is a major cause of community-acquired pneumonia and invasive pneumococcal disease, particularly in older adults and people with cardiovascular disease. Pneumococcal infection may precipitate heart failure, arrhythmias, myocardial infarction, stroke, functional decline, hospitalization, and death. This review examines pneumococcal disease as a cardiovascular stressor, the effectiveness of adult pneumococcal vaccination, and the comparative clinical relevance of higher-valency conjugate vaccines. **Methods:** We conducted a narrative review of randomized trials, phase 3 immunogenicity and safety studies, real-world effectiveness analyses, observational cardiovascular cohorts, meta-analyses, epidemiological surveillance reports, regulatory documents, and current European, U.S., and Spanish recommendations. **Results:** Clinical evidence supports protection against vaccine-type invasive pneumococcal disease and pneumococcal pneumonia. Associations between pneumococcal vaccination and lower myocardial infarction risk derive predominantly from observational studies and do not establish causality; a reduction in cardiovascular events remains unproven. The 23-valent polysaccharide vaccine is an older, unconjugated platform that induces a T-cell-independent response without the immunological memory produced by conjugate vaccines; therefore, a higher nominal valency does not necessarily imply greater or more durable clinical effectiveness. PCV20 and PCV21 are higher-valency conjugate vaccines with partially overlapping non-nested serotype compositions. Both have adult regulatory indications in Spain, but their evidence bases differ: PCV20 has phase 3 immunogenicity and safety evidence plus emerging real-world effectiveness data, whereas PCV21 currently has a broad phase 3 immunogenicity and safety program. No head-to-head clinical-outcome study has established superior effectiveness of either vaccine. Differences in estimated serotype coverage reflect epidemiological overlap and do not directly quantify clinical protection. Spanish serotype epidemiology is highly dynamic, with persistent adult disease caused by serotypes included in each formulation and recent increases in selected legacy serotypes retained in PCV20. A serotype-specific association between serotypes 3 and 9N and cardiovascular events has been reported in one invasive-disease cohort but was not reproduced in a larger pneumococcal pneumonia cohort. **Conclusions:** Pneumococcal vaccination should be integrated into cardiovascular care for eligible adults to prevent pneumococcal disease. Any additional benefit in preventing myocardial infarction or other cardiovascular events, including long-term complications, remains uncertain. Vaccine selection should follow official recommendations and consider age, previous vaccination, regional epidemiology, serotype-associated disease burden and severity, antimicrobial resistance, and availability rather than valency or immunogenicity alone.

## 1. Introduction

In cardiovascular care, residual risk is usually framed in terms of low-density lipoprotein cholesterol, blood pressure, glycemia, thrombosis, obesity, kidney dysfunction, smoking, and heart failure progression. This framework is incomplete because acute respiratory infections frequently destabilize otherwise stable chronic cardiovascular disease. Pneumococcal pneumonia is a relevant example: it is common, potentially severe, partly preventable by vaccination, and disproportionately affects older adults and those with multimorbidity.

The consequences of pneumococcal infection extend beyond the lungs. Community-acquired pneumonia (CAP) and invasive pneumococcal disease (IPD) can provoke hypoxemia, systemic inflammation, endothelial dysfunction, platelet activation, autonomic stress, myocardial oxygen supply-demand mismatch, arrhythmias, heart failure decompensation, acute kidney injury, and death. Experimental and translational studies also support pneumolysin-mediated myocardial injury, microvascular invasion, and subsequent remodeling in severe pneumococcal disease [1,2]. These mechanisms provide a compelling rationale for including vaccination in cardiovascular prevention. Nevertheless, one distinction is essential throughout this review: prevention of vaccine-type pneumococcal disease is supported by direct efficacy and effectiveness evidence, whereas evidence for the prevention of major adverse cardiovascular events (MACE) remains indirect and mainly observational.

Cardiovascular guidance has recently repositioned adult vaccination from an ancillary task to an integral component of preventive care. The 2025 European Society of Cardiology (ESC) clinical consensus statement and the 2026 American College of Cardiology (ACC) guidance both support systematic immunization assessment in patients with cardiovascular disease [3,4]. In parallel, adult pneumococcal policy has evolved rapidly with simplified schedules and the availability of higher-valency conjugate vaccines, particularly PCV20 and PCV21 [5,6,7].

This review examines the association between pneumococcal disease and cardiovascular complications, critically evaluates the evidence on vaccination in adults, compares PCV20 and PCV21 using the same regulatory, immunological, clinical, and epidemiological domains, and proposes practical pathways for implementation in cardiology. Particular attention is paid to avoiding two common errors: equating vaccine valency with clinical effectiveness and treating a cross-sectional estimate of serotype coverage as a fixed ranking of vaccines.

## 2. Objectives and Scope

This narrative review addresses six objectives:

1. To summarize the evidence linking pneumococcal pneumonia and invasive infection to acute cardiovascular destabilization.
2. To review the efficacy and effectiveness of adult pneumococcal vaccination for IPD, pneumococcal pneumonia, all-cause pneumonia, hospitalization, and mortality.
3. To discuss the specific cardiovascular relevance of pneumococcal vaccination in adults with established heart disease.
4. To synthesize current guidance from cardiovascular societies, public-health authorities, and regulatory agencies.
5. To compare PCV20 and PCV21 in a balanced manner, including adult authorization, serotype composition, immunogenicity, safety, clinical effectiveness, and epidemiological trade-offs.
6. To translate the evidence into implementable pathways and measurable quality indicators for cardiovascular practice.

The review is clinically oriented and mirrors the practical logic used for influenza vaccination in cardiovascular prevention while adapting the strength of each statement to the pneumococcal evidence base. It is not a systematic review or meta-analysis; quantitative estimates are therefore presented descriptively and interpreted according to study design, comparator, population, endpoint, and baseline risk.

## 3. Evidence Selection and Conceptual Framework

This narrative review examined pneumococcal disease, adult pneumococcal vaccination, and their relevance to cardiovascular care. The initial literature search was conducted on 1 July 2026, followed by a targeted update on 24 August 2026. Publications and official documents available by the latter date were considered. Sources included PubMed, encompassing MEDLINE-indexed and other PubMed records, ClinicalTrials.gov, and the official websites of regulatory agencies, public-health authorities, and professional societies. During manuscript revision, the cited vaccination recommendations and policies were rechecked against official CDC/ACIP, ESC, ACC, Spanish Ministry of Health, and Castilla y León sources on 17 October 2026. This targeted verification of guidance and policy documents was conducted separately from the clinical literature search. A further targeted check of PCV21 composition, immunogenicity, and serotype-associated outcomes was conducted during revision on 19 October 2026, including the ERS 2025 conference abstract discussed below.

Eligible clinical publications concerned adults aged ≥ 18 years and addressed at least one of the following: cardiovascular complications associated with pneumonia or invasive pneumococcal disease; pneumococcal vaccine immunogenicity, safety, efficacy, or effectiveness; or serotype distributions relevant to adult vaccination. Outcomes of interest included invasive disease, pneumonia, hospitalization, mortality, and cardiovascular events. Randomized trials, observational studies, systematic reviews, and meta-analyses were considered. Selected experimental studies were included to explain biological mechanisms, while pediatric evidence was considered only when relevant to indirect protection or changes in adult serotype epidemiology. Publications were considered irrespective of the direction or statistical significance of their findings.

Regulatory information was obtained from the European Medicines Agency and the Spanish Agency of Medicines and Medical Devices, including product information available through CIMA. Vaccination recommendations were obtained from CDC/ACIP, Spanish national and regional public-health authorities, and relevant cardiovascular societies. Documents were selected according to their issuing authority, applicability to adult vaccination, jurisdiction, and version available by the evidence cutoff. Surveillance reports were selected for their relevance to adult disease burden, serotype distribution, and antimicrobial resistance, with attention to the reporting period, geographical coverage, age groups, and completeness of serotyping. ClinicalTrials.gov records were used to clarify trial design and publication status; registration alone was not treated as evidence of clinical benefit.

PCV20 and PCV21 were appraised using the same domains: regulatory indication and dose, serotype composition, immunogenicity, safety, clinical effectiveness, evidence limitations, potential age- and region-specific coverage, and the severity and prognosis of disease associated with the included serotypes. Differences in opsonophagocytic activity (OPA) responses were not interpreted as evidence of superior clinical effectiveness in the absence of comparative clinical-outcome data. Likewise, potential serotype coverage was defined as the proportion of observed disease attributable to serotypes included in a formulation; it was not treated as synonymous with vaccine effectiveness.

**Figure 1:**
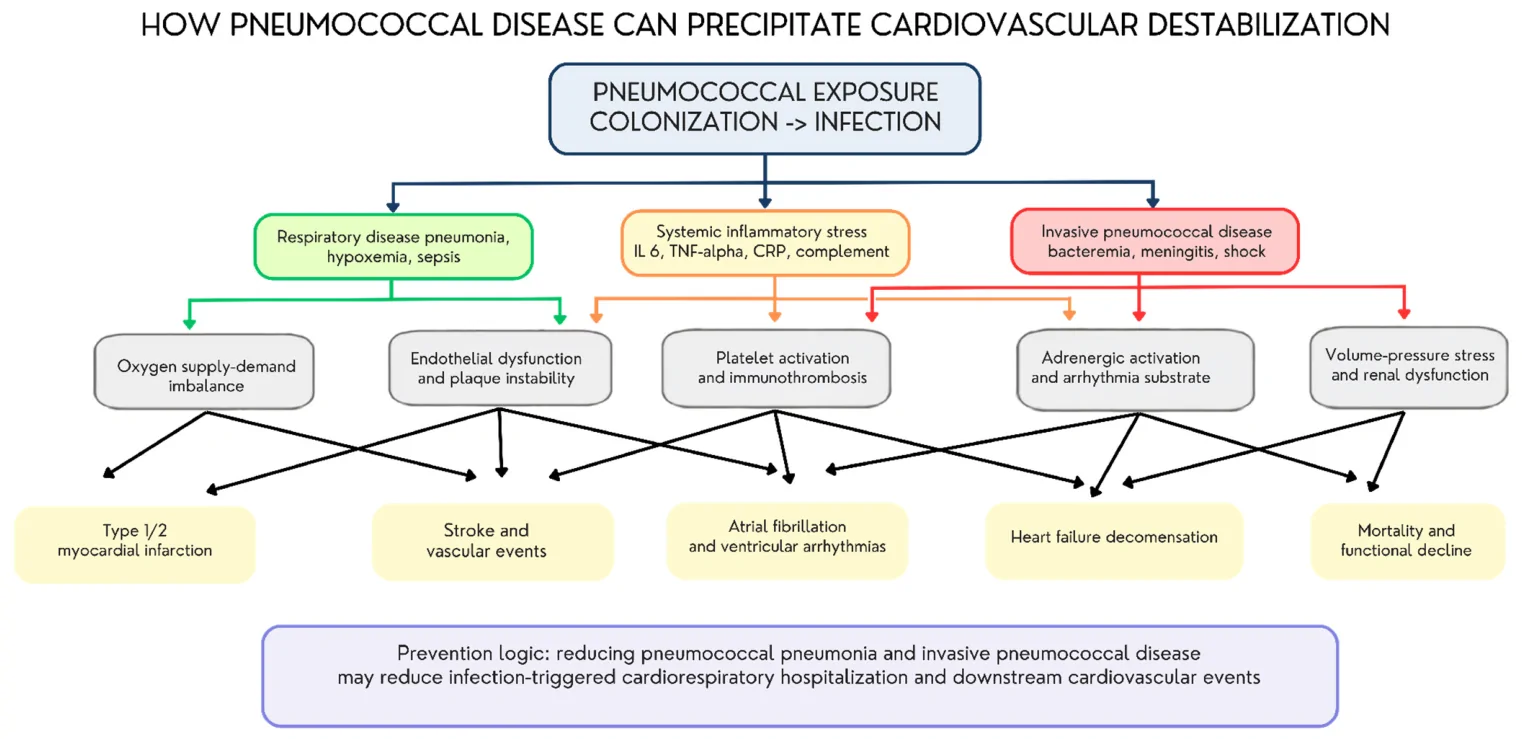
Pathophysiologic pathways linking pneumococcal disease with cardiovascular destabilization. Green-, orange-, and red-outlined boxes represent respiratory disease, systemic inflammatory stress, and invasive pneumococcal disease, respectively. Gray boxes indicate intermediate pathophysiological mechanisms, pale yellow boxes indicate clinical outcomes, and the purple-outlined box summarizes the rationale for prevention. Colored arrows correspond to pathways originating from boxes outlined in the same color; black arrows link intermediate mechanisms to potential clinical outcomes. CRP, C-reactive protein; IL, interleukin; TNF, tumor necrosis factor.

The evidence was organized around four clinical questions: (1) Does pneumococcal disease act as a cardiovascular trigger? (2) Do available vaccines prevent pneumococcal disease and severe pneumonia? (3) Does vaccination translate into fewer cardiovascular events? (4) How should cardiology teams implement vaccination given the varying strength of the supporting evidence? (Figure 1).

## 4. Benefits of Pneumococcal Vaccination in the General Adult Population

The primary rationale for pneumococcal vaccination is prevention of vaccine-type IPD and pneumococcal pneumonia. Reducing these infections may limit triggers of cardiovascular destabilization, but prevention of subsequent cardiovascular events, including long-term complications, remains unproven. In Spain, national surveillance recorded 5362 autochthonous IPD cases in 2024, with a notification rate approximately 75% higher than in 2016. However, the national report attributes part of this increase to improvements in surveillance completeness and more comprehensive access to clinical microbiology laboratory data. This increase in notification rates should therefore not be interpreted as an equivalent increase in the underlying incidence of IPD [8]. Mortality was concentrated in older adults, and the overall case-fatality proportion among cases with a known outcome was 13% [8].

**Figure 2:**
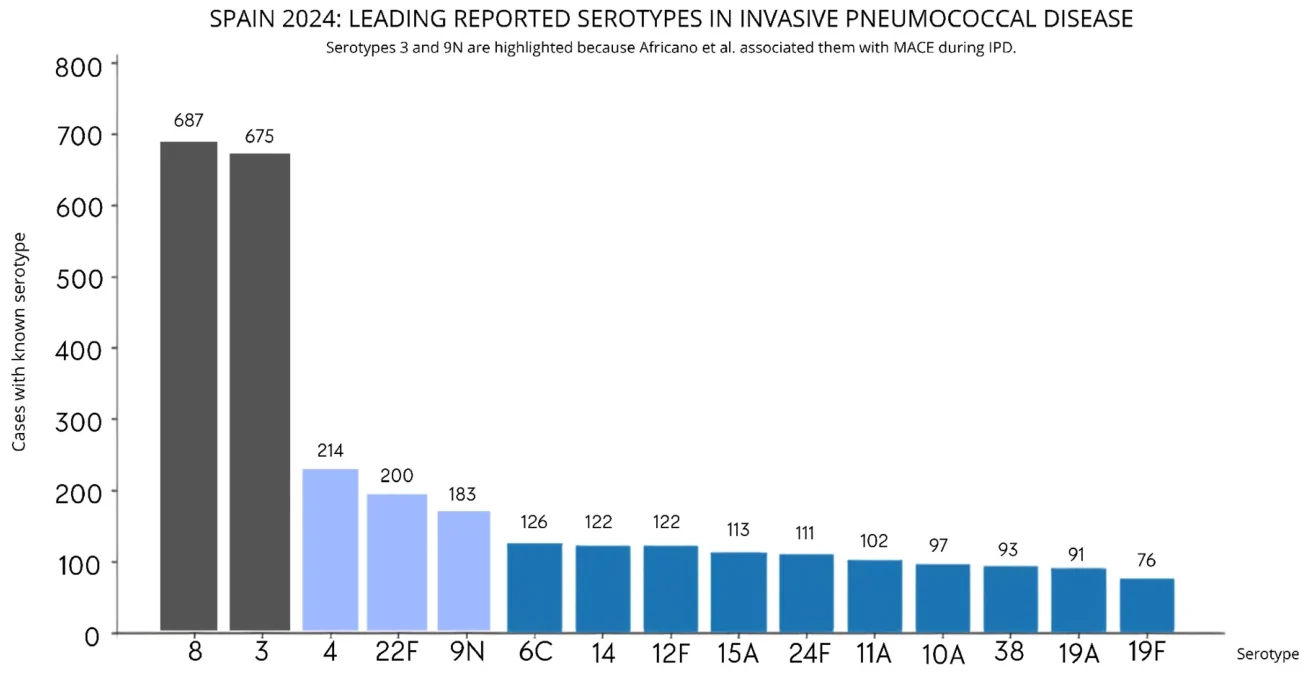
Leading serotypes among invasive pneumococcal disease cases with known serotype in Spain, 2024. Dark gray bars represent serotypes 8 and 3; light blue bars represent serotypes 4, 22F, and 9N; and blue bars represent the remaining serotypes. The colors are used for visual differentiation and do not indicate differences in cardiovascular risk or disease severity. Serotypes 3 and 9N are highlighted because they were independently associated with MACE in one retrospective IPD cohort; this highlighting does not imply that they are the only or predominant contributors to severe pneumococcal disease, hospitalization, or mortality [9].

The serotype was reported for 3836 cases (72% of notifications), and 67 different serotypes were identified. Five serotypes accounted for approximately half of serotyped cases: 8 (687 cases; 17.9%), 3 (675; 17.6%), 4 (214; 5.6%), 22F (200; 5.2%), and 9N (183; 4.8%) [8]. This distribution illustrates that the adult disease burden is distributed across serotypes with different frequencies, invasive potential, resistance profiles, and clinical associations [9]. Frequency, lethality, invasiveness, and cardiovascular risk are related but non-interchangeable dimensions; a serotype associated with MACE is not necessarily the leading cause of CAP, IPD, hospitalization, or death (Figure 2) [9].

CAPiTA was a randomized, double-blind, placebo-controlled trial involving 84,496 adults aged ≥ 65 years, with a mean follow-up of 3.97 years. In the per-protocol analysis, PCV13 efficacy against a first episode of vaccine-type pneumococcal CAP was 45.6% (95.2% CI, 21.8–62.5); efficacy against nonbacteremic/noninvasive vaccine-type pneumococcal CAP was 45.0% (95.2% CI, 14.2–65.3), and efficacy against vaccine-type IPD was 75.0% (95% CI, 41.4–90.8). In the modified intention-to-treat analysis, efficacy against all-cause CAP was 5.1% (95% CI, −5.1 to 14.2), without a statistically significant reduction. These findings establish efficacy against vaccine-type pneumococcal disease; the trial was not designed to evaluate cardiovascular outcomes [10].

PPSV23 is an older, unconjugated 23-valent polysaccharide vaccine. It provides broad nominal serotype coverage and established protection against vaccine-type IPD, but evidence for noninvasive or all-cause pneumonia and mortality has been less consistent, particularly in adults with chronic disease [11]. Its capsular polysaccharides induce a predominantly T-cell-independent response; unlike conjugate vaccines, PPSV23 does not generate the same serotype-specific memory B-cell response or anamnestic response. Antibody levels may decline within 3–5 years and sometimes earlier in older or otherwise vulnerable populations [12,13]. Thus, the relevant distinction is not that PPSV23 is less serotype-specific, but that its immune response is generally less robust and less durable and lacks the immunological memory produced by conjugation.

Accordingly, vaccine valency, defined as the number of serotypes included, should not be used as a surrogate for clinical effectiveness. Clinical value also depends on the platform, the magnitude and persistence of the immune response, the epidemiological relevance of the included serotypes and their associations with disease severity and prognosis, cross-protection, baseline risk, and the endpoint evaluated.

Higher-valency conjugate strategies address the need for durable protection in adults while simplifying schedules. PCV20 is supported by phase 3 immunogenicity and safety studies in vaccine-naive and previously vaccinated adults, as well as emerging real-world effectiveness data in older adults [14,15,16]. PCV21 is supported by phase 3 immunogenicity and safety studies in vaccine-naive, previously vaccinated, at-risk, and HIV-positive adults [17,18,19,20,21]. These evidence bases should be compared by endpoint and maturity, while also considering the frequency, severity, and prognosis of disease associated with the included serotypes, rather than valency alone (Table 1).

## 5. Specific Benefits of Pneumococcal Vaccination in People with Cardiovascular Disease

Adults with cardiovascular disease have two overlapping vulnerabilities. First, chronic heart disease is a recognized risk condition for severe pneumococcal disease. Second, pneumonia and invasive infection expose the cardiovascular system to abrupt hypoxemic, inflammatory, thrombotic, autonomic, and hemodynamic stress. The resulting clinical trajectory may include heart failure decompensation, acute coronary syndromes, arrhythmias, stroke, acute kidney injury, delirium, functional decline, and death [1,22].

Acute cardiac complications are frequent during hospitalization for CAP. Cohorts have documented new or worsening heart failure, arrhythmias, and myocardial infarction, often early after admission and with adverse prognostic implications [23,24]. Hospitalization for pneumonia is also associated with an increased subsequent cardiovascular risk that extends beyond the acute episode [25]. This association describes the prognosis following pneumonia and should not be interpreted as evidence that pneumococcal vaccination prevents these subsequent events or their long-term consequences. In severe pneumococcal disease, experimental and translational data support pneumolysin-mediated myocardial injury, bacterial translocation to cardiac tissue, microvascular involvement, and later remodeling [2].

Cardiovascular meta-analyses have reported associations between pneumococcal vaccination and selected clinical outcomes. Marra et al. included 18 studies, involving 716,108 adults, that evaluated PPSV23 against study-specific controls, including no vaccination, placebo, or another vaccine. Pooled risk ratios were 0.91 (95% CI, 0.84–0.99) for any cardiovascular event, as defined in the contributing studies, 0.88 (95% CI, 0.79–0.98) for myocardial infarction, and 0.78 (95% CI, 0.68–0.88) for all-cause mortality. No statistically significant reduction in cerebrovascular disease was observed [26]. Jaiswal et al. pooled 15 observational studies involving 347,444 adults with or without established cardiovascular disease, comparing pneumococcal vaccination with no pneumococcal vaccination. Vaccination was associated with lower all-cause mortality (HR, 0.76; 95% CI, 0.66–0.87) and a lower risk of myocardial infarction (HR, 0.73; 95% CI, 0.56–0.96), with substantial heterogeneity (I^2^ = 90% and 84%, respectively). Associations with cardiovascular mortality (HR, 0.87; 95% CI, 0.72–1.07) and stroke (HR, 1.01; 95% CI, 0.93–1.10) were not statistically significant. Insufficient information prevented analysis by vaccine formulation [27]. These pooled associations remain vulnerable to residual confounding, healthy-user bias, and differences in populations, vaccination histories, and outcome definitions. They should not be interpreted as proof of cardiovascular protection or extrapolated directly to PCV20 or PCV21. A population-based cohort has also suggested possible differences between sequential and single pneumococcal vaccination strategies, but such findings cannot substitute for randomized cardiovascular-outcome trials [28].

Randomized evidence is now available from AUSPICE, which evaluated PPSV23 versus placebo in adults aged 55–60 years with cardiovascular risk factors but no previous cardiovascular event. The trial found no statistically significant reduction in its primary cardiovascular outcome (HR, 0.90; 95% CI, 0.63–1.28), although fewer events than anticipated limited statistical power. These findings do not establish cardiovascular protection and should not be directly extrapolated to conjugate vaccines or secondary-prevention populations [29].

Serotype-specific epidemiology requires particular care. Multiple serotypes contribute to IPD, hospitalization, antimicrobial resistance, and death through different combinations of frequency and virulence. Africano et al. reported MACE in 23% of 310 adults with IPD and identified serotypes 3 and 9N as independent correlates of MACE after adjustment for clinical factors [9]. This finding is relevant but limited in scope: it does not establish that these serotypes account for most pneumococcal pneumonia, invasive disease, hospitalization, severe disease, or mortality, and it does not prove that vaccination against them prevents MACE.

The serotype-specific cardiovascular signal has not been consistently reproduced. In a larger Spanish cohort of 1739 episodes of pneumococcal CAP, acute cardiac events occurred in 17.5% of episodes, but no individual serotype or clonal complex was significantly associated with those events. Older age, pre-existing heart disease, bacteremia, septic shock, and high-risk pneumonia were the principal independent predictors [30]. Accordingly, the association of serotypes 3 and 9N with MACE should be regarded as hypothesis-generating rather than as a general hierarchy of serotype severity.

Serotype 14 illustrates why overall disease burden and associations with cardiovascular events must be separated. It is not consistently identified as an independent clinical predictor of cardiovascular events, and the principal experimental myocardial-injury models do not establish a serotype-14-specific mechanism. Nevertheless, serotype 14 remains clinically relevant because it can cause invasive disease, has shown renewed circulation in Spanish adults, and has been linked to penicillin non-susceptibility in recent regional surveillance [8,31]. Thus, a vaccine appraisal should integrate frequency, invasiveness, antimicrobial resistance, lethality, and emerging cardiovascular signals rather than prioritizing a single serotype or endpoint.

Pneumococcal vaccination should be recommended to eligible adults on the basis of its established role in preventing pneumococcal disease. Observational associations with lower myocardial infarction risk remain susceptible to residual confounding and healthy-user bias and do not establish a causal effect. Prevention of cardiovascular events should therefore be described as a potential additional benefit requiring further confirmation (Table 2).

## 6. Relevant Guidelines and Regulatory Context

The 2026 ACC Concise Clinical Guidance recommends pneumococcal vaccination for adults aged ≥ 50 years who have not previously received a pneumococcal conjugate vaccine and for adults aged 19–49 years with qualifying chronic conditions, including chronic heart disease, heart failure, and cardiomyopathy. Hypertension alone does not constitute a risk-based indication. Depending on previous vaccination, eligible adults may receive PCV20 or PCV21, or PCV15 followed by PPSV23 [4,5,6,7].

Together with the 2025 ESC consensus, the updated ACC guidance supports integrating vaccination into routine cardiovascular care [3,4]. Implementation should include a review of vaccination history, discussion of patient concerns and preferences, a documented recommendation, and vaccine administration or coordinated referral when on-site vaccination is unavailable. Follow-up should confirm completion of the recommended schedule.

For adults in Spain, PCV20 and PCV21 are both authorized for active immunization to prevent invasive disease and pneumonia caused by *S. pneumoniae* in persons aged 18 years or older. Each is administered as a single 0.5-mL intramuscular dose in adults and should be used according to official recommendations; there is no established requirement for routine revaccination [13,32]. Regulatory authorization should not be confused with preferential recommendation, public funding, or universal interchangeability: the product used in practice depends on age, previous vaccination, clinical risk, regional policy, and availability.

Spanish adult pneumococcal vaccination is implemented through life-course and risk-group strategies that may differ among autonomous communities [33]. Regional programs already illustrate age- and risk-specific use of the two higher-valency conjugate options; for example, one 2026 regional program selected PCV21 for older adults and institutionalized adults while retaining PCV20 for specified risk groups at younger ages [34]. Cardiologists should therefore avoid reproducing a single fixed schedule in discharge reports and instead use electronic prompts linked to the current regional calendar (Table 3).

## 7. Higher-Valency Pneumococcal Conjugate Vaccines in Adults: PCV20 and PCV21

PCV20 and PCV21 are complementary higher-valency conjugate strategies rather than successive versions of the same serotype set. Their compositions overlap only partially: PCV21 is not PCV20 plus one serotype. Each formulation contains serotypes not present as nominal components in the other, and the relative epidemiological value of those differences changes by age, region, and time. Consequently, neither valency nor a single immunogenicity comparison establishes superior overall clinical effectiveness.

### 7.1. PCV20: Composition, Evidence, Safety, and Serotype Rationale

PCV20 contains capsular polysaccharides from serotypes 1, 3, 4, 5, 6A, 6B, 7F, 8, 9V, 10A, 11A, 12F, 14, 15B, 18C, 19A, 19F, 22F, 23F, and 33F, individually conjugated to CRM197. In adults, it is authorized in Spain for active immunization against invasive disease and pneumonia caused by *S. pneumoniae*, with a single 0.5-mL dose administered according to official recommendations [13].

The pivotal phase 3 study enrolled pneumococcal vaccine-naive adults aged 18 years or older. Among adults aged 60 years or older, PCV20 met the prespecified non-inferiority criteria versus PCV13 for all 13 shared serotypes and versus PPSV23 for 6 of the 7 additional serotypes; serotype 8 narrowly missed the statistical criterion, although a functional immune response was elicited. Robust responses were observed for all 20 vaccine serotypes across age groups, and the safety profile was comparable with those of the control vaccines [14].

A separate phase 3 study in adults aged 65 years or older previously vaccinated with PPSV23, PCV13, or a sequential PCV13-PPSV23 schedule found that PCV20 elicited functional responses against all 20 serotypes regardless of the previous regimen and was generally well tolerated [15]. This evidence is particularly relevant to real-world cardiology populations with heterogeneous and sometimes incompletely documented vaccination histories.

The clinical effectiveness of PCV20 was evaluated in a retrospective cohort of 16,503,552 U.S. Medicare beneficiaries aged ≥ 65 years, followed between July 2022 and June 2024. The analysis compared PCV20-vaccinated with PCV20-unvaccinated person-time, applying prespecified restrictions on previous pneumococcal vaccination. Outcomes were defined as the first recorded diagnoses identified using ICD-10-CM codes. Adjusted vaccine effectiveness, calculated as (1 − adjusted HR) × 100%, was 25.6% (95% CI, 17.4–33.0) against IPD due to any serotype, 23.3% (95% CI, 16.3–29.8) against pneumococcal pneumonia due to any serotype, 15.2% (95% CI, 14.5–15.9) against all-cause pneumonia, and 7.4% (95% CI, 6.9–7.8) against lower respiratory tract infection [16]. These observational estimates are based on coded clinical diagnoses rather than immunogenicity or laboratory-confirmed vaccine-type endpoints and remain susceptible to diagnostic misclassification and residual confounding. The study did not assess cardiovascular outcomes.

A clinically important feature of PCV20 is its retention of several legacy serotypes that are absent as nominal components from PCV21, including 1, 4, 5, 6B, 9V, 14, 18C, 19F, and 23F. Some of these serotypes became less common following pediatric conjugate vaccination, but recent surveillance has documented renewed circulation of selected members of this group, particularly serotypes 4 and 14 in younger and middle-aged adults [8,31]. These observations inform the epidemiological relevance of PCV20’s serotype composition but do not demonstrate greater clinical efficacy or effectiveness than PCV21 in these populations (Table 4).

### 7.2. PCV21: Composition, Evidence, Safety, and Serotype Rationale

PCV21 contains capsular polysaccharides from serotypes 3, 6A, 7F, 8, 9N, 10A, 11A, 12F, 15A, 15C (represented by de-O-acetylated 15B polysaccharide), 16F, 17F, 19A, 20A, 22F, 23A, 23B, 24F, 31, 33F, and 35B, conjugated to CRM197. The de-O-acetylated 15B polysaccharide has a molecular structure similar to that of 15C; immune responses to this component are assessed using serotype 15C, while responses to serotype 15B are cross-reactive. The number 21 denotes vaccine valency and must not be confused with pneumococcal serotype 21 [32].

In STRIDE-3, vaccine-naive adults received PCV21 or PCV20. PCV21 met non-inferiority criteria for the 10 serotypes common to both vaccines and superiority criteria for 10 of the 11 additional serotypes in PCV21; superiority was not demonstrated for 15C. Safety profiles were broadly similar [17]. These results establish comparative immunogenicity, not superior protection against IPD, pneumonia, hospitalization, mortality, or cardiovascular events.

The wider phase 3 program evaluated previously vaccinated adults aged 50 years or older (STRIDE-6), vaccine-naive adults aged 50 years or older using PPSV23 as the comparator (STRIDE-10), people living with HIV (STRIDE-7), and adults aged 18–64 years with chronic conditions that increased the risk of pneumococcal disease, including heart disease, diabetes, and renal, hepatic, or pulmonary disease (STRIDE-8) [18,19,20,21]. Across these populations, PCV21 elicited robust immune responses and was generally well tolerated. Clinical-effectiveness data against IPD, pneumococcal pneumonia, hospitalization, mortality, or cardiovascular outcomes have not yet been published.

PCV21 includes 11 serotypes that are absent as nominal components from PCV20: 9N, 15A, 15C, 16F, 17F, 20A, 23A, 23B, 24F, 31, and 35B. These serotypes contribute to a substantial burden of adult pneumococcal disease, particularly in older adults [8,32]. The adult-specific rationale for PCV21 therefore encompasses both the frequency of disease caused by these serotypes and its clinical consequences, including severe infection and cardiovascular complications. These considerations inform the epidemiological and clinical relevance of its serotype composition (Table 4).

An observational study presented at ERS 2025 included 859 pneumococcal vaccine-naive, immunocompetent adults hospitalized with bacteremic pneumococcal pneumonia at four Spanish hospitals. Disease caused by the PCV21 serotype group was associated with higher rates of new-onset atrial fibrillation and in-hospital mortality [35]. This conference abstract supports considering serotype-associated prognosis alongside disease frequency. However, the analysis concerned the overall PCV21 serotype group, not specifically its 11 additional serotypes relative to PCV20. These preliminary associations do not establish uniform excess severity across those 11 serotypes or comparative vaccine effectiveness.

### 7.3. Dynamic Serotype Epidemiology and the Clinical Trade-Off Between PCV20 and PCV21

Potential serotype coverage is an epidemiological estimate: the proportion of cases with an identified serotype attributable to serotypes included in a vaccine, in a specified population and period, for a defined clinical endpoint. It does not measure vaccine efficacy or effectiveness and should not be interpreted as the proportion of cases that vaccination would prevent. Clinical protection also depends on serotype-specific vaccine performance, host characteristics, previous vaccination, and duration of protection. Moreover, coverage estimates derived from IPD surveillance cannot be directly extrapolated to noninvasive pneumonia or cardiovascular outcomes. Higher estimated serotype coverage therefore does not, by itself, establish superior clinical protection.

The possibility that classic serotypes omitted from a new adult formulation could persist or re-emerge if direct vaccine pressure decreases is biologically plausible and clinically important. However, it remains a surveillance hypothesis rather than a demonstrated consequence of PCV21 use. Population-level pressure against these serotypes may continue through pediatric schedules and indirect protection, while local transmission, under-vaccination, antimicrobial resistance, and clonal expansion may act in the opposite direction.

Spanish surveillance demonstrates that the balance is not fixed. In the 2024 national report, serotype 4 was the third most frequent serotype among people aged 15–64 years and had increased consistently during the preceding three years [8]. In a 2007–2025 population-based study conducted in the Community of Madrid among adults aged 18–59 years, the most prevalent serotypes in 2023–2025 were 8, 3, 4, and 14. Compared with 2015–2019, incidence increased markedly in 2025 for serotypes 14, 9V, and 4; serotypes 14 and 9V were associated with penicillin non-susceptibility [31]. These serotypes included in PCV20 can increase the relative potential coverage of PCV20 in younger adult populations and specific regions.

Conversely, PCV21 contains non-PCV20 serotypes that contribute substantially to the adult disease burden, including 9N, 15A, 23A, 23B, 24F, 31, and 35B. In older adults, the collective burden of these serotypes may favor PCV21 in terms of potential serotype coverage in many current datasets [8]. The exact difference has changed over recent years and should be recalculated rather than assumed. National surveillance age bands also do not permit an exact 18–59-year estimate, so regional findings should not be automatically generalized to all of Spain.

These observations have three practical implications. First, PCV20 and PCV21 should not be ordered hierarchically by valency. Second, the difference between contemporary coverage estimates may narrow or reverse as serotype distribution changes. Third, vaccine selection should follow current national or regional recommendations and be reassessed using age-stratified surveillance, antimicrobial resistance patterns, previous vaccination, product availability, and emerging clinical-effectiveness data. No randomized or observational head-to-head clinical-outcome study has established the superiority of either PCV20 or PCV21 for IPD, pneumococcal pneumonia, hospitalization, mortality, or MACE.

## 8. Practical Use of Pneumococcal Vaccines in Patients with Cardiovascular Disease

Recommendations for pneumococcal vaccination in cardiovascular practice should follow applicable age- and risk-based guidance and be grounded in the prevention of pneumococcal disease. Preventing infection may reduce triggers of cardiovascular destabilization, but a reduction in myocardial infarction, stroke, or other cardiovascular events has not been established.

The favorable cardiovascular associations reported in observational studies and meta-analyses remain susceptible to residual confounding, healthy-user bias, and differences in healthcare access, frailty, smoking, and adherence to preventive treatment [26,27]. These findings should not be presented as proof of cardiovascular protection. Vaccination should form part of comprehensive preventive care alongside guideline-directed cardiovascular treatment.

Pneumococcal vaccination strategies should also be distinguished from post-myocardial-infarction influenza vaccination. Influenza trials support vaccination before discharge after an acute coronary syndrome; equivalent evidence from randomized trials of pneumococcal vaccination after acute coronary syndrome is not available. Nevertheless, hospitalization, discharge after hospitalization for heart failure, evaluation for structural heart disease, visits to device clinics, cardiac rehabilitation, and routine follow-up are high-yield opportunities to assess eligibility and prior PCV/PPSV23 vaccination.

**Figure 3:**
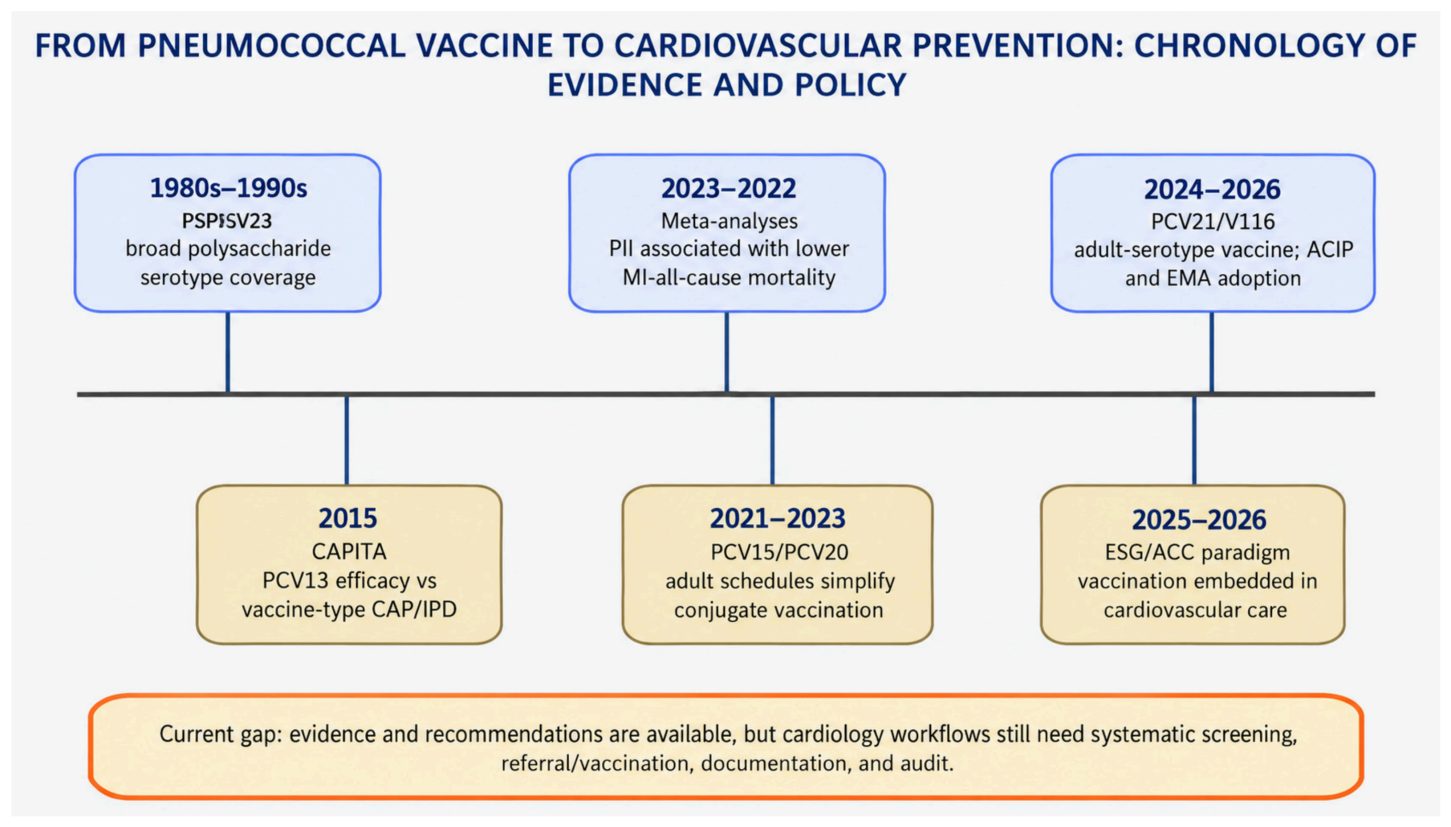
Chronology of adult pneumococcal vaccine evidence and policy relevant to cardiovascular prevention.

Once the patient is clinically stable and has no relevant contraindication, the cardiovascular team should make an explicit recommendation and either administer the vaccine or activate a same-day referral pathway. At a minimum, the clinical record should document eligibility, previous vaccination, the recommended next step, where administration will occur, and how completion will be confirmed. Deferral without assigning responsibility for follow-up commonly results in a missed opportunity for prevention (Figure 3).

## 9. Next Steps and Future Directions: Time for Implementation

**Figure 4:**
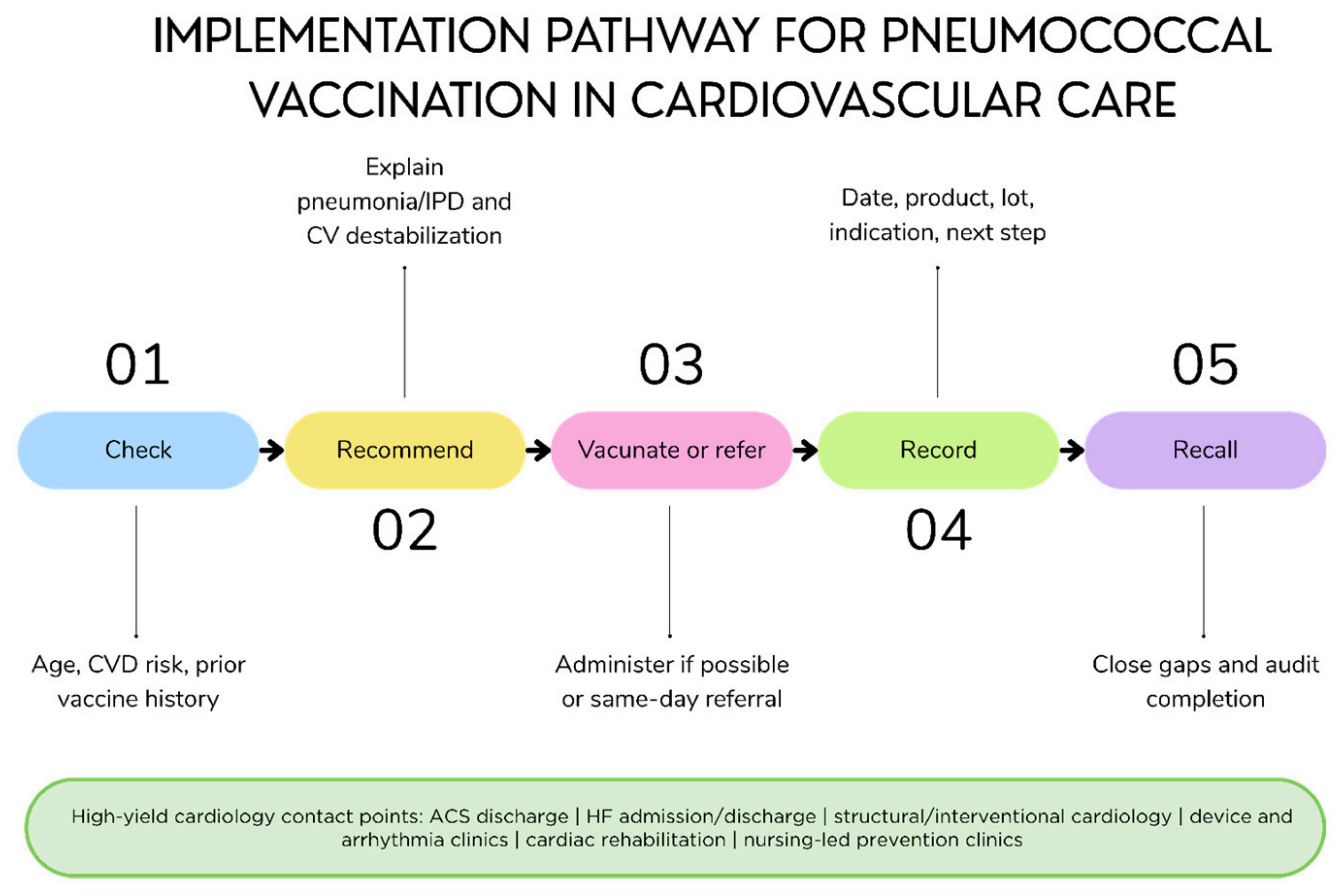
Practical pathway to integrate pneumococcal vaccination into cardiovascular care. ACS, acute coronary syndrome; CV, cardiovascular; CVD, cardiovascular disease; HF, heart failure; IPD, invasive pneumococcal disease.

The main barrier has shifted from a lack of recommendations to inconsistent implementation. Pneumococcal vaccination should become a visible and auditable variable in cardiovascular care. A minimum pathway includes five actions: check eligibility and history, recommend, vaccinate or refer, record, and recall. These actions should be embedded in electronic and clinical workflows rather than left to individual memory (Figure 4).

**Figure 5:**
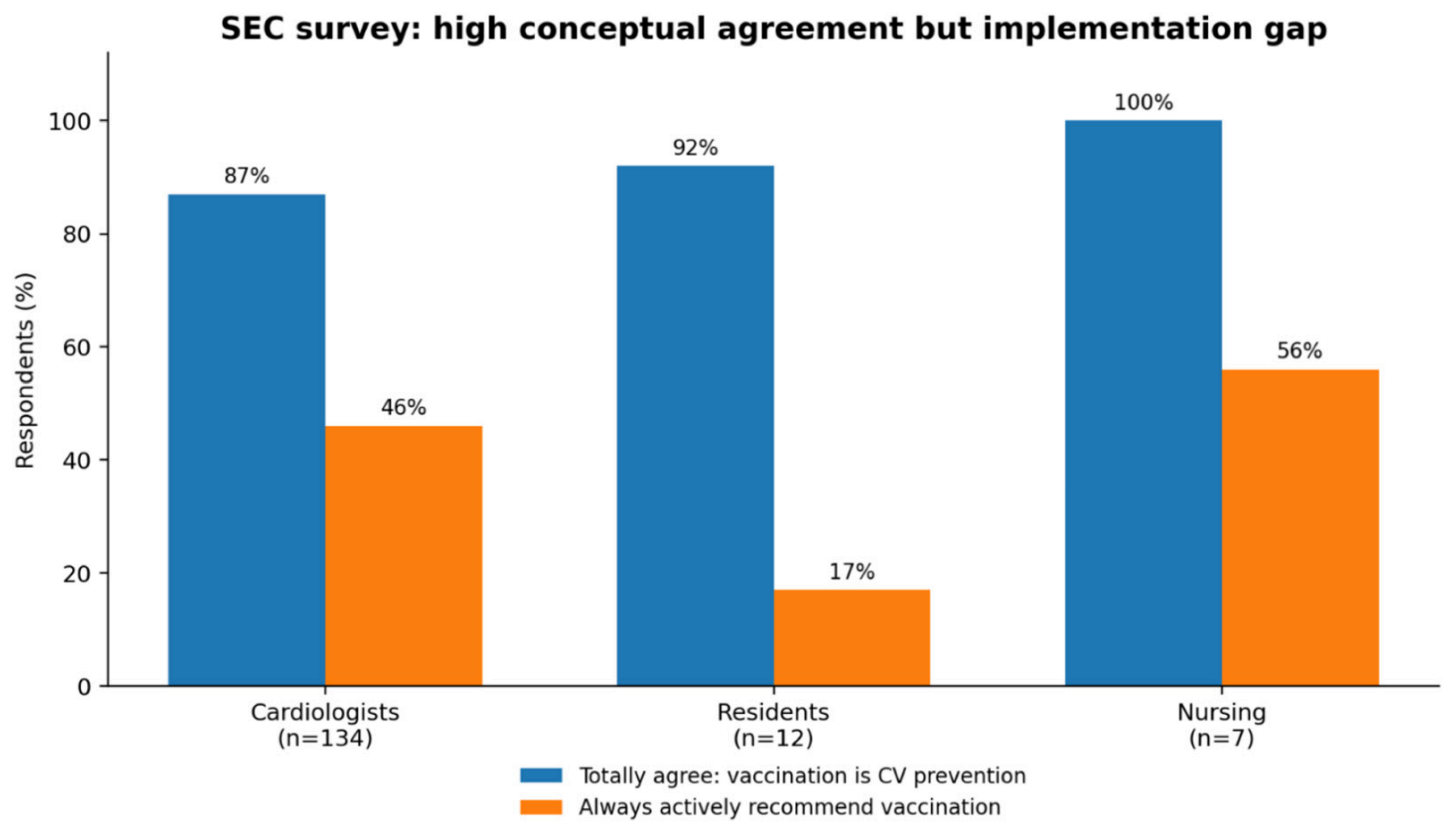
Attitudes toward vaccination as cardiovascular prevention and self-reported active recommendation: preliminary conference-reported survey findings [36]. Group sizes are shown below each professional category. CV, cardiovascular; SEC, Spanish Society of Cardiology.

Preliminary findings from an online survey of healthcare professionals linked to the Spanish Society of Cardiology suggest a possible gap between attitudes toward vaccination and self-reported recommendation practices [36]. Although 87% of cardiologists, 92% of residents, and 100% of nursing respondents agreed that vaccination is a form of cardiovascular prevention, only 46%, 17%, and 56%, respectively, reported always actively recommending vaccination. Pneumococcal vaccination was consistently identified as one of the priority vaccines. Reported barriers included limited time, insufficient training, uncertainty about the evidence, patient reluctance, and coordination problems with primary care [36] (Figure 5). These observations should be interpreted cautiously because of the small, unequal groups of professional respondents and potential selection and nonresponse bias. They should not be interpreted as representative estimates of vaccination practices among Spanish healthcare professionals.

Implementation should therefore focus on workflow redesign: electronic health record prompts, structured vaccine fields in discharge templates, vaccination review within heart failure and rehabilitation programs, nursing- or pharmacist-supported pathways, and closed-loop referral with primary care, preventive medicine, pharmacies, and public health. A recommendation that is not documented cannot be audited, and a referral that is not closed frequently fails (Table 5 and Table 6).

Future research should prioritize: (1) comparative real-world effectiveness of PCV20 and PCV21 in age- and risk-stratified cardiovascular populations; (2) effects on vaccine-type IPD, pneumococcal pneumonia, cardiorespiratory hospitalization, and antimicrobial-resistant disease; (3) prospective assessment of cardiovascular outcomes after vaccination in high-risk patients; (4) post-implementation surveillance of legacy-serotype persistence or re-emergence; and (5) implementation trials of electronic alerts, discharge bundles, pharmacist-led services, and cardiology-nursing protocols.

## 10. Limitations of the Available Evidence

The evidence linking pneumococcal vaccination with cardiovascular prevention is heterogeneous. Some studies evaluate vaccine-type IPD, whereas others evaluate pneumococcal pneumonia, all-cause pneumonia, lower respiratory tract infection, hospitalization, mortality, or cardiovascular endpoints. These outcomes are related but not interchangeable. Vaccine-type IPD and vaccine-type pneumococcal pneumonia are the most specific outcomes for assessing pneumococcal vaccine efficacy; all-cause pneumonia, cardiorespiratory hospitalization, MACE, and mortality are more vulnerable to endpoint dilution and confounding.

Evidence suggesting cardiovascular benefit from pneumococcal vaccination is predominantly observational and remains susceptible to residual confounding and healthy-user bias [26,27]. Randomized evidence is limited. In AUSPICE, PPSV23 versus placebo in adults aged 55–60 years with cardiovascular risk factors but no previous cardiovascular event did not significantly reduce the primary composite of acute coronary syndrome and ischemic stroke (HR, 0.90; 95% CI, 0.63–1.28). The lower-than-expected event rate limited statistical power [29]. A causal cardiovascular benefit therefore remains unproven.

The PCV20 and PCV21 evidence bases also differ in maturity. PCV20 has phase 3 immunogenicity and safety data together with emerging retrospective effectiveness evidence in older adults; PCV21 currently has an extensive phase 3 immunogenicity and safety program but no published studies of clinical effectiveness. No head-to-head clinical-outcome study has established comparative effectiveness against IPD, pneumococcal pneumonia, hospitalization, mortality, or cardiovascular events. Serotype-associated severity and prognosis are also relevant to vaccine appraisal, but observational associations do not establish the clinical benefits of a particular formulation. Differences in OPA cannot be translated directly into differences in clinical effectiveness, particularly for nonbacteremic pneumonia, for which no universally accepted correlate of protection exists [13,32].

Estimates of potential serotype coverage are also limited. They depend on data from cases with a known serotype, may use age bands that do not match regulatory or clinical groups, and do not incorporate serotype-specific vaccine effectiveness, cross-protection, severity, lethality, antimicrobial resistance, indirect effects, or future serotype replacement. Spanish epidemiology is changing rapidly, and regional findings may not represent the whole country. Product-level comparisons should therefore be updated continuously rather than treated as permanent.

Finally, this narrative review did not use independent screening by two reviewers, formal risk-of-bias assessment, or quantitative pooling. Although the search strategy, eligibility criteria, and approach to selecting gray literature are described, the synthesis remains subject to selection bias and may have omitted relevant evidence. Regulatory and surveillance sources also differ in scope, reporting completeness, and update frequency. The review should therefore complement formal guidelines and current local public-health recommendations.

## 11. Conclusions

Pneumococcal vaccination has a generally favorable safety profile in the studied adult populations. Prevention of vaccine-type invasive disease and pneumococcal pneumonia is established. Preventing pneumococcal disease may reduce infectious triggers of cardiovascular destabilization; however, prevention of subsequent cardiovascular events and long-term cardiovascular complications has not been established.

A broader direct cardioprotective effect is biologically plausible, and observational studies and meta-analyses suggest possible reductions in myocardial infarction and all-cause mortality. However, this evidence is weaker than the evidence for infection prevention and should be described accordingly. The association of serotypes 3 and 9N with MACE in one IPD cohort is an important hypothesis-generating signal, not proof that these serotypes dominate overall pneumococcal severity or that their inclusion in a vaccine reduces MACE. A larger pneumococcal CAP cohort found no independent serotype association, emphasizing the importance of host factors, bacteremia, shock, and pneumonia severity [9,30].

PPSV23 remains historically important but is an older unconjugated platform with a T-cell-independent, less durable immune response and no conjugate-vaccine immunological memory. Its 23-valent composition does not imply inherently greater effectiveness than that of a lower-valency conjugate vaccine. PCV20 and PCV21 are both relevant adult conjugate options with partially overlapping non-nested serotype compositions. PCV20 retains several classic serotypes whose renewed circulation may matter in younger adults and specific regions; PCV21 includes 11 serotypes absent as nominal components from PCV20 that contribute to a substantial burden of adult pneumococcal disease, particularly in older adults. Their frequency and the severity and prognosis of the disease they cause are relevant to vaccine appraisal. Vaccine valency, immunogenicity, and estimated serotype coverage do not, by themselves, establish comparative clinical efficacy or effectiveness.

Vaccine selection should therefore follow current national or regional recommendations and consider age, clinical risk, previous vaccination, regional serotype distribution, serotype-associated severity and prognosis, antimicrobial resistance, product availability, and emerging effectiveness evidence. The immediate priority is implementation: assess, recommend, vaccinate or refer, document, recall, and audit vaccination delivery across cardiology, primary care, nursing, rehabilitation, pharmacy, preventive medicine, and long-term care.

## Figures and Tables

**Table 1:** Summary of pneumococcal vaccine evidence in the general adult population.

Vaccine/Evidence Base	Design or Comparator	Main Results	Interpretation
PPSV23	Randomized and observational evidence; unconjugated polysaccharide platform.	Established protection against vaccine-type IPD; less consistent evidence for non-invasive or all-cause pneumonia and mortality. T-cell-independent response without conjugate-vaccine immunological memory.	Historically important broad-valency vaccine. Its nominal valency does not imply greater clinical effectiveness or durability than a conjugate vaccine [11,12].
PCV13/CAPiTA	Randomized, double-blind trial of PCV13 versus placebo in 84,496 adults aged ≥ 65 years; mean follow-up, 3.97 years.	Per-protocol efficacy against first episodes: vaccine-type pneumococcal CAP, 45.6% (95.2% CI, 21.8–62.5); nonbacteraemic/non-invasive vaccine-type CAP, 45.0% (95.2% CI, 14.2–65.3); vaccine-type IPD, 75.0% (95% CI, 41.4–90.8). Efficacy against all-cause CAP in the modified intention-to-treat analysis was 5.1% (95% CI, −5.1 to 14.2).	Randomized evidence of efficacy against vaccine-type pneumococcal disease. No statistically significant reduction in all-cause CAP. Cardiovascular outcomes were not evaluated [10].
PCV20/pivotal phase 3 immunogenicity trial	Randomized immunogenicity and safety trial in vaccine-naive adults aged ≥ 18 years. Non-inferiority comparisons in adults aged ≥ 60 years used PCV13 for shared serotypes and PPSV23 for additional serotypes.	Non-inferiority criteria were met for all 13 serotypes shared with PCV13 and 6 of 7 additional serotypes compared with PPSV23. Serotype 8 narrowly missed the prespecified criterion, although a functional immune response was elicited.	Supports immunogenicity and safety. These immune-response endpoints do not directly quantify protection against clinical disease or cardiovascular events [14].
PCV20/Medicare effectiveness study	Retrospective cohort of 16,503,552 U.S. adults aged ≥ 65 years, July 2022–June 2024. PCV20-vaccinated versus PCV20-unvaccinated person-time, with restrictions on prior pneumococcal vaccination. First diagnoses identified using ICD-10-CM codes.	Adjusted effectiveness: any-serotype IPD, 25.6% (95% CI, 17.4–33.0); any-serotype pneumococcal pneumonia, 23.3% (95% CI, 16.3–29.8); all-cause pneumonia, 15.2% (95% CI, 14.5–15.9); lower respiratory tract infection, 7.4% (95% CI, 6.9–7.8).	Observational clinical-effectiveness estimates based on coded diagnoses, without vaccine-type confirmation. Residual confounding and diagnostic misclassification remain possible. Cardiovascular outcomes were not assessed [16].
PCV21 *	Phase 3 immunogenicity and safety program across vaccine-naive, previously vaccinated, at-risk, and HIV-positive adults.	Non-inferior OPA responses for 10 serotypes common to PCV20 and superior responses for 10 of 11 additional PCV21 serotypes; superiority was not demonstrated for 15C.	Broad phase 3 evidence, but clinical effectiveness against IPD, pneumonia, hospitalization, mortality, or cardiovascular events remains to be established [17,18,20,21].

* PCV21 includes serotype 15C, represented by de-O-acetylated 15B polysaccharide, and elicits cross-reactive immune responses to serotype 15B. Confidence intervals are reported at the 95% level, except for the two CAPiTA vaccine-type pneumonia endpoints, for which the original publication reported 95.2% confidence intervals. Estimates should not be compared directly across studies because populations, comparators, outcome definitions, and study designs differ. CAP, community-acquired pneumonia; CAPiTA, Community-Acquired Pneumonia Immunization Trial in Adults; CI, confidence interval; HIV, human immunodeficiency virus; IPD, invasive pneumococcal disease; OPA, opsonophagocytic activity; PCV13, 13-valent pneumococcal conjugate vaccine; PCV20, 20-valent pneumococcal conjugate vaccine; PCV21, 21-valent pneumococcal conjugate vaccine; PPSV23, 23-valent pneumococcal polysaccharide vaccine.

**Table 2:** Key studies linking pneumococcal disease and vaccination to cardiovascular outcomes.

Study	Population/Design	Key Cardiovascular Finding	Interpretation
Musher et al., 2007 [23]	Adults with pneumococcal pneumonia	Association between pneumococcal pneumonia and acute cardiac events.	Identifies pneumococcal infection as a cardiac trigger [23].
Corrales-Medina et al., 2012 [24]	Hospitalized CAP cohort	Acute cardiac complications occurred early and were associated with short-term mortality.	Supports early monitoring and prevention of severe pneumonia [24].
Corrales-Medina et al., 2015 [25]	Pneumonia hospitalization and subsequent cardiovascular disease	Pneumonia was associated with increased subsequent cardiovascular risk.	Identifies increased cardiovascular risk after pneumonia; does not establish that vaccination prevents subsequent cardiovascular events.
Africano et al., 2021 [9]	310 adults with serotyped IPD	MACE occurred in 23%; serotypes 3 and 9N were independently associated with MACE.	Serotype-specific signal that is clinically relevant but hypothesis-generating [9].
Rombauts et al., 2020 [30]	1739 pneumococcal CAP episodes; 872 serotyped isolates	No significant association between individual serotypes and acute cardiac events; host factors, bacteraemia, shock, and pneumonia severity predicted events.	Important counterpoint to a fixed serotype hierarchy [30].
Marra et al., 2020 [26]	Systematic review and meta-analysis of 18 studies involving 716,108 adults. PPSV23 versus study-specific controls, including no vaccination, placebo, or another vaccine.	Pooled RR: any cardiovascular event, as defined in the contributing studies, 0.91 (95% CI, 0.84–0.99); myocardial infarction, 0.88 (95% CI, 0.79–0.98); all-cause mortality, 0.78 (95% CI, 0.68–0.88). No statistically significant reduction in cerebrovascular disease.	Associations concern PPSV23 and heterogeneous clinical endpoints. They do not establish a causal cardiovascular benefit or provide formulation-specific evidence for PCV20 or PCV21 [26].
Hure et al., 2025 [29]	Placebo-controlled PPSV23 trial; primary prevention.	No significant reduction in the primary cardiovascular outcome.	Limited power; applicability to conjugate vaccines and secondary prevention remains uncertain [29]
Jaiswal et al., 2022 [27]	Systematic review and meta-analysis of 15 observational studies involving 347,444 adults with or without established cardiovascular disease. Pneumococcal vaccination versus no pneumococcal vaccination.	Pooled HR: myocardial infarction, 0.73 (95% CI, 0.56–0.96; I^2^ = 84%); all-cause mortality, 0.76 (95% CI, 0.66–0.87; I^2^ = 90%). No statistically significant association with cardiovascular mortality, 0.87 (95% CI, 0.72–1.07), or stroke, 1.01 (95% CI, 0.93–1.10).	Observational associations with substantial heterogeneity for myocardial infarction and all-cause mortality. Residual confounding limits causal inference; insufficient information prevented analysis by vaccine formulation [27].

CAP, community-acquired pneumonia; CI, confidence interval; HR, hazard ratio; IPD, invasive pneumococcal disease; MACE, major adverse cardiovascular events; RR, risk ratio.

**Table 3:** Guidelines, regulatory conditions, and public-health recommendations relevant to cardiovascular practice.

Source	Target Population	Pneumococcal Strategy	Cardiology Implication
CDC/ACIP	Adults ≥ 50 years; adults aged 19–49 years with specified risk conditions, including chronic heart disease.	PCV20 or PCV21 may serve as single-dose conjugate options in eligible adults; PCV15 followed by PPSV23 is an alternative depending on history.	Simplifies identification and permits cardiology-triggered vaccination or referral [5,6,7].
ACC 2026	Adults ≥ 50 years without previous PCV; adults aged 19–49 years with qualifying chronic conditions, including chronic heart disease.	PCV20 or PCV21, or PCV15 followed by PPSV23, according to vaccination history.	Integrate vaccination assessment, counselling, administration or referral, and follow-up into cardiovascular care [4].
ESC 2025	Patients with established cardiovascular disease or high cardiovascular risk.	Integrates vaccination against respiratory pathogens into cardiovascular prevention.	Supports systematic assessment during routine cardiovascular encounters [3].
Spain/regional programs	Older adults and risk groups; age thresholds and vaccine selection vary by autonomous community.	PCV20 and PCV21 are adult single-dose conjugate options used according to regional policy and prior vaccination.	Requires current local-calendar knowledge and coordination with primary care and public health [33,34].

ACC, American College of Cardiology; ACIP, Advisory Committee on Immunization Practices; CDC, Centers for Disease Control and Prevention; ESC, European Society of Cardiology; PCV15, 15-valent pneumococcal conjugate vaccine; PCV20, 20-valent pneumococcal conjugate vaccine; PCV21, 21-valent pneumococcal conjugate vaccine; PPSV23, 23-valent pneumococcal polysaccharide vaccine.

**Table 4:** PCV20 and PCV21: serotype composition and immunogenicity evidence.

Domain	PCV20	PCV21
Serotype composition	1, 3, 4, 5, 6A, 6B, 7F, 8, 9V, 10A, 11A, 12F, 14, 15B, 18C, 19A, 19F, 22F, 23F, and 33F [13].	3, 6A, 7F, 8, 9N, 10A, 11A, 12F, 15A, 15C (de-O-acetylated 15B), 16F, 17F, 19A, 20A, 22F, 23A, 23B, 24F, 31, 33F, and 35B [32].
Pivotal immunogenicity findings	Met non-inferiority criteria versus PCV13 for all 13 shared serotypes and versus PPSV23 for 6 of 7 additional serotypes; serotype 8 did not meet the prespecified criterion [14].	Met non-inferiority criteria versus PCV20 for 10 shared serotypes and superiority criteria for 10 of 11 additional PCV21 serotypes; superiority was not demonstrated for 15C [17].
Additional populations studied	Adults previously vaccinated with PPSV23, PCV13, or sequential PCV13–PPSV23 [15].	Previously vaccinated adults, adults with chronic risk conditions, and people living with HIV [18,20,21].

Both vaccines use CRM197 as the carrier protein. The de-O-acetylated 15B component of PCV21 represents serotype 15C because of their similar polysaccharide structures and elicits cross-reactive immune responses to serotype 15B. Differences in serotype composition, estimated serotype coverage, or immunogenicity do not establish comparative clinical efficacy or effectiveness. Clinical efficacy and effectiveness findings are summarized separately in Table 1. A reduction in cardiovascular events has not been established for either vaccine. PCV13, 13-valent pneumococcal conjugate vaccine; PCV20, 20-valent pneumococcal conjugate vaccine; PCV21, 21-valent pneumococcal conjugate vaccine; PPSV23, 23-valent pneumococcal polysaccharide vaccine.

**Table 5:** Practical pathway to integrate pneumococcal vaccination across cardiovascular care settings.

Care Setting	Trigger Point/Action	Rationale
Acute coronary syndrome discharge	Review pneumococcal status before discharge or at early follow-up; vaccinate or refer when stable and eligible.	Prevents a missed opportunity and creates a documented plan.
Heart failure admission/discharge	Include vaccine status in transition planning.	High absolute risk of pneumonia-triggered decompensation and readmission.
Outpatient cardiology	Review adult vaccination status at least annually or during major therapeutic reassessment.	Pairs immunization with medication optimization and risk-factor review.
Cardiac rehabilitation	Use a vaccination checklist at intake and verify completion.	Provides structured education and longitudinal contact.
Arrhythmia/device clinics	Use recurrent visits and anticoagulation reviews to identify gaps.	High contact frequency facilitates recall and documentation.
Structural/interventional cardiology	Review status during pre-procedural assessment and after stabilization.	Captures older, multimorbid adults at high infection-related risk.
Primary care/pharmacy pathway	Use same-day referral or direct administration according to local policy.	Reduces fragmentation between recommendation and completion.
Long-term care	Audit vaccination status on admission and periodically.	Frailty, institutionalization, and outbreak risk increase absolute benefit.

**Table 6:** Suggested quality indicators for pneumococcal vaccination in cardiovascular care.

Indicator Type	Metric	Purpose
Process	Percentage of eligible cardiovascular patients with documented pneumococcal vaccination status.	Measures whether vaccination is visible in the cardiovascular record.
Process	Percentage of eligible unvaccinated patients receiving an explicit recommendation or same-day referral.	Measures clinical action rather than passive awareness.
Process	Percentage of heart failure or acute coronary syndrome discharge reports containing vaccination status and a plan.	Targets high-risk transitions of care.
Outcome/process	Percentage of referred patients with confirmed vaccination completion within 30–90 days.	Measures closure of the care loop.
Equity	Coverage stratified by age, sex, socioeconomic proxy, region, institutionalization, and comorbidity.	Detects inequities in access and implementation.
Clinical outcome	Rates of pneumococcal pneumonia, IPD, all-cause pneumonia, cardiorespiratory hospitalization, heart failure readmission, and mortality.	Supports quality improvement; causal inference requires appropriate study designs.

IPD, invasive pneumococcal disease.

## Data Availability

The original contributions presented in this study are included in the article. Further inquiries can be directed to the corresponding authors.

## Abbreviations

The following abbreviations are used in this manuscript:

ACC	American College of Cardiology
ACIP	Advisory Committee on Immunization Practices
ACS	Acute coronary syndrome
CAP	Community-acquired pneumonia
CDC	Centers for Disease Control and Prevention
CRM197	Non-toxic mutant of diphtheria toxin used as a carrier protein
CVD	Cardiovascular disease
EHR	Electronic health record
ESC	European Society of Cardiology
HF	Heart failure
IPD	Invasive pneumococcal disease
MACE	Major adverse cardiovascular events
MI	Myocardial infarction
OPA	Opsonophagocytic activity
PCV	Pneumococcal conjugate vaccine
PPSV23	23-valent pneumococcal polysaccharide vaccine
